# Overwhelming Gradient of Aortic Stenosis in the Context of Dysthyroidism: A Case Report

**DOI:** 10.7759/cureus.90767

**Published:** 2025-08-22

**Authors:** Mouad Lamtai, Loubna Elkhadir, Hajar Moukane, Nora Bennani, Rokia Fellat, Nadia Fellat

**Affiliations:** 1 Cardiology, Centre Hospitalo-Universitaire Ibn Sina, Rabat, MAR; 2 Cardiology Center, Mohamed V Military Hospital, Rabat, MAR

**Keywords:** aortic stenosis, hemodynamics, high gradient, hyperthyroidism, reversible gradient

## Abstract

Thyroid hormones significantly influence the heart's metabolism, playing a key role in regulating cardiac function and cardiovascular hemodynamics. In cases of thyrotoxicosis, the excessive production of thyroid hormones induces remarkable hemodynamic alterations in the cardiovascular system. In this report, we discuss the case of a 70-year-old patient admitted for dyspnea with a severe high gradient of aortic stenosis due to an overactive thyroid. This case report aims to illustrate the impact of systemic conditions on cardiac hemodynamics.

## Introduction

Hyperthyroidism is a medical condition characterized by the excessive production and release of thyroid hormones by the thyroid gland. The cardiovascular manifestations range from sinus tachycardia to atrial fibrillation, from a high-output cardiac state to chronic heart failure. The correction of hyperthyroidism is associated with improvement in cardiac hemodynamics, with some case reports in the literature describing reversible cases of severe tricuspid and mitral regurgitation [1,2].

We present the case of a 70-year-old patient admitted with dyspnea due to a severe high-gradient aortic stenosis secondary to hyperthyroidism. This report illustrates the potential of hyperthyroidism in exacerbating aortic stenosis and highlights the importance of hemodynamic evaluation in patients presenting with overwhelming valvular heart disease parameters. 

## Case presentation

A 70-year-old woman with a history of hypertension, managed with calcium channel blockers, and dyslipidemia, on simvastatin, presented to our facility with worsening exertional dyspnea (New York Heart Association (NYHA) class II).

**Figure 1:**
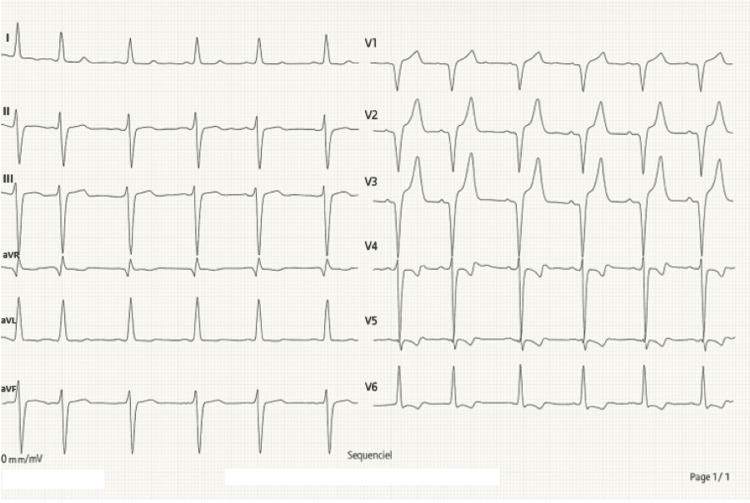
EKG showing left ventricular hypertrophy with strain pattern and incomplete left bundle branch block

At presentation, her blood pressure was 118/85 mmHg with a heart rate of 90 beats/minute. Physical exam showed a systolic murmur at the right upper sternal border. The EKG demonstrated left ventricular hypertrophy with a strain pattern and incomplete left bundle branch block (Figure 1).

**Figure 2:**
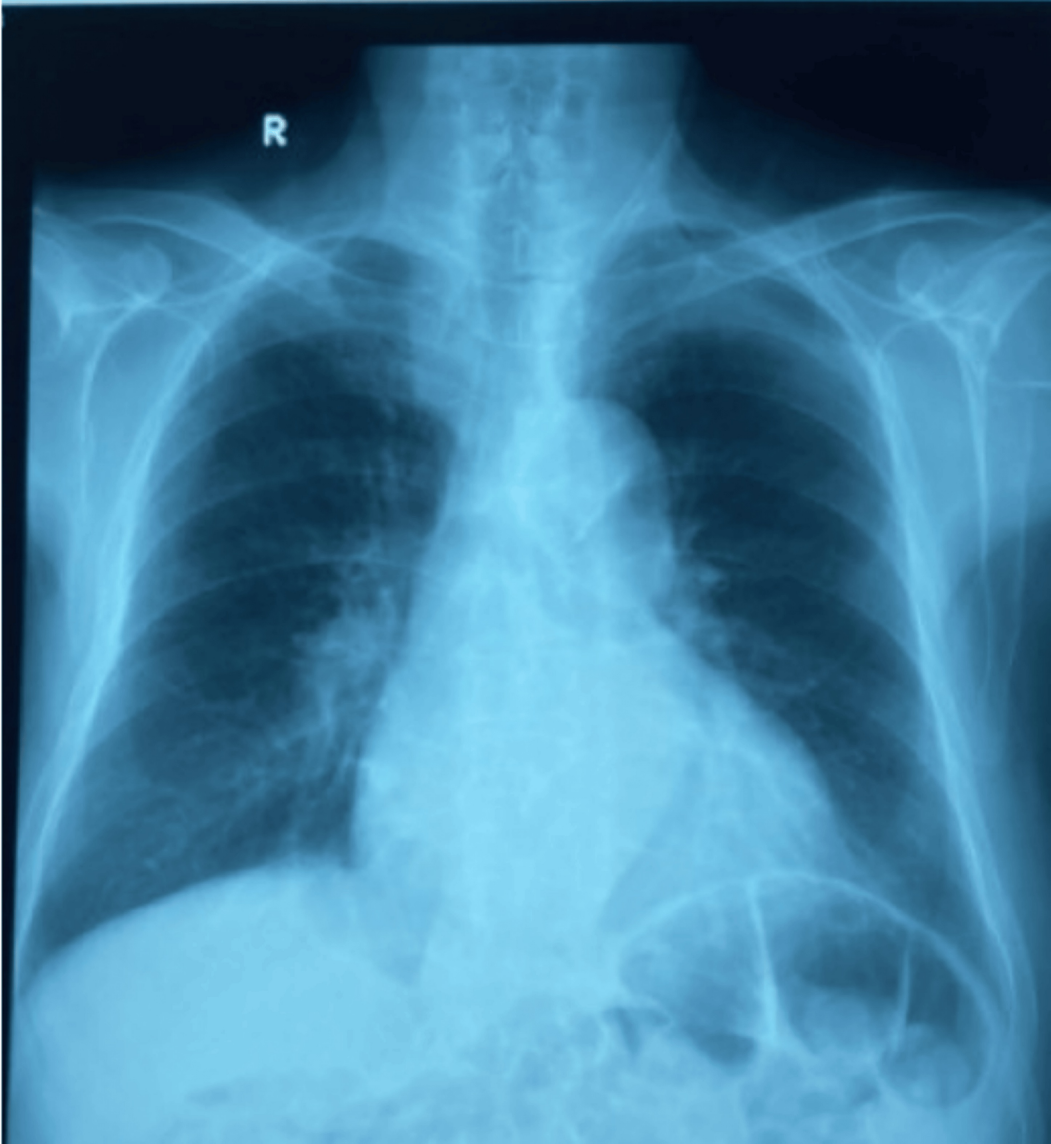
Chest X-ray showing cardiomegaly with calcification of the aortic borders

**Figure 3:**
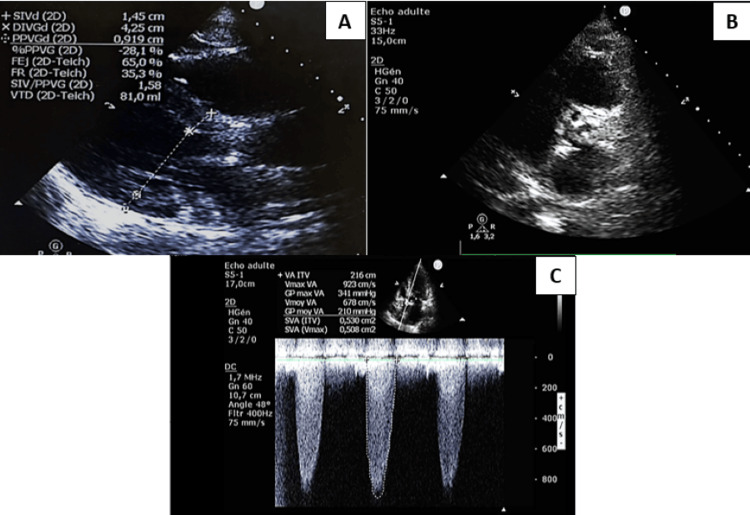
Transthoracic echocardiography showing concentric left ventricular hypertrophy(A) and a heavily calcified aortic valve (B) with overwhelming aortic stenosis parameters (C)

Chest X-ray demonstrated an enlarged cardiac silhouette with calcification of the aortic borders (Figure 2). Transthoracic echocardiography showed a heavily calcified aortic valve with a huge aortic stenosis parameter with left ventricular concentric hypertrophy (thickness of the septum wall was 14 mm), the aortic systolic flow was estimated to be 9 m/second, and the mean aortic gradient has reached 210 mmHg with an aortic valve area: 0.53 cm^2^. Left ventricular ejection fraction was 52% with no left ventricular wall motion abnormality. The atria and aortic root were not dilated (Figure 3).

The coronary angiogram showed calcified and atheromatous coronary arteries: tight focal lesion of the mid circumflex artery, tight lesion at the first bend of the right coronary artery, followed by an intermediate lesion of the second segment of the right coronary artery (RCA II), long 30% stenosis of the proximal to mid left anterior descending artery (LAD). 

The hormonal assessment revealed hyperthyroidism; TSH was low at 0.142 mUI/L (reference range:0.4-4 mUI/L), T4 was 2 mUI/L (reference range: 0.70-1.4 mUI/L), and the anti-thyroid peroxidase and anti-thyroglobulin antibodies were negative. A thyroid ultrasound was performed, which was normal.

**Figure 4:**
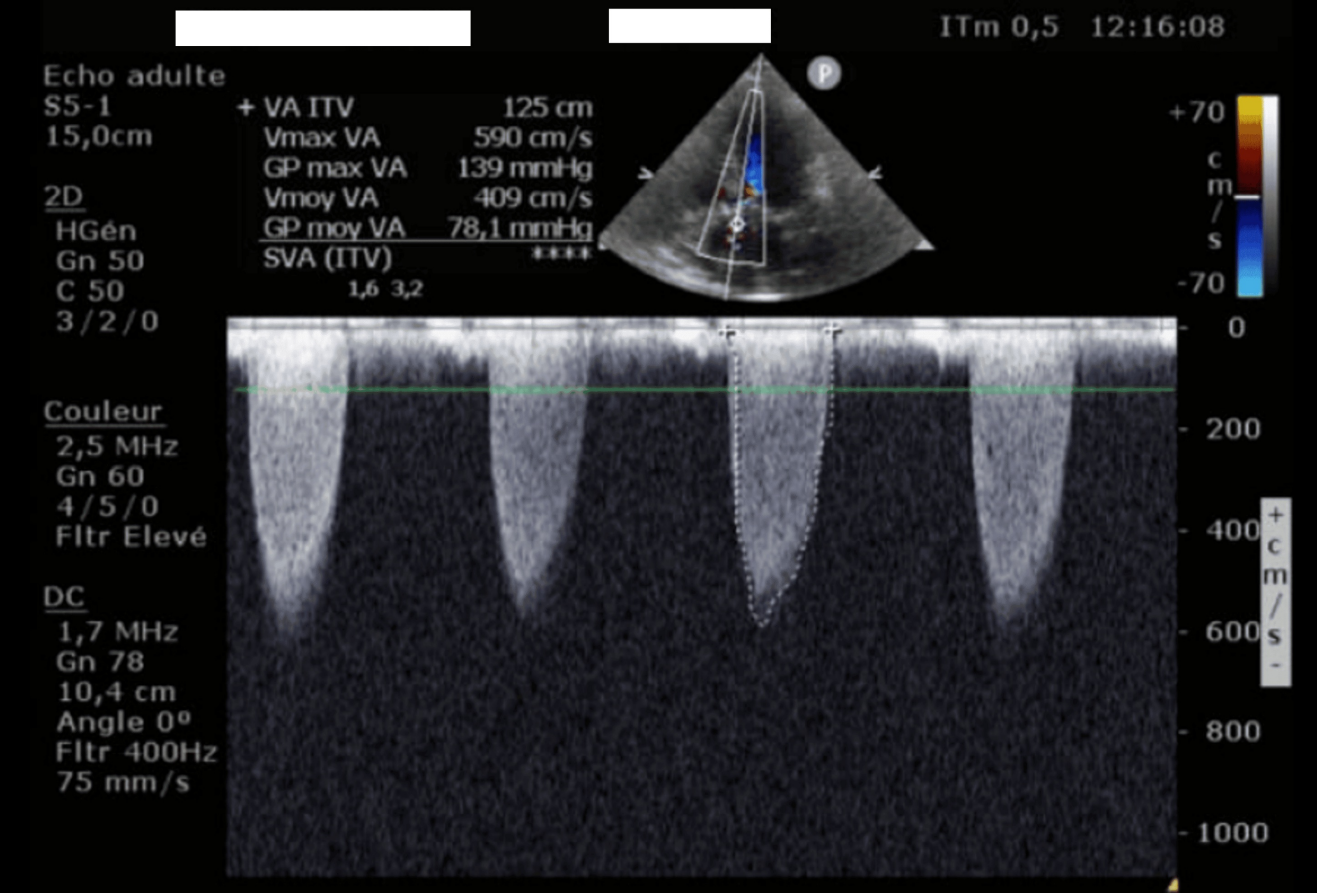
Apical five-chamber view showing a regression in the aortic stenosis parameters

Synthetic antithyroid therapy was initiated using carbimazole 10 mg. Thyroid parameters normalized within four weeks, and echocardiographic follow-up showed a marked reduction in the aortic gradient to 78.1 mmHg and peak transvalvular velocity to 5.90 m/second (Figure 4).

The patient underwent aortic valve replacement along with coronary artery bypass graft surgery. Following cardiac surgery, the patient no longer experienced dyspnea, and the two-month postprocedure transthoracic echocardiography showed normal left ventricular function with normal function of the aortic prosthetic.

## Discussion

Aortic stenosis is one of the most common valvular heart diseases and involves a narrowing of the aortic valve opening, which limits blood flow from the left ventricle to the aorta. It is mostly caused by rheumatic heart disease or calcific degeneration in the elderly [3].

This case report presents an unusual scenario where the gradient of the aortic stenosis was exacerbated by an underlying hyperthyroidism. The severity of the aortic stenosis appeared disproportionate to the echocardiographic valve anatomy, suggesting a functional component to the obstruction. Following treatment and normalizing of thyroid function, the aortic stenosis gradient has improved, emphasizing the dynamic interplay between thyroid function and cardiovascular state.

Several pathological mechanisms may explain the observed findings. Thyrotoxicosis leads to a hyperdynamic and hyperkinetic cardiovascular state by increasing heart rate and preload, which potentially increases the transvalvular flow and the aortic valve gradient [4,5]. Hyperthyroidism is also associated with a higher coronary calcification index, an increased incidence of atherosclerotic cardiac events, and greater cardiovascular mortality, which are attributed to endothelial damage, thrombosis, and hemodynamic changes [6].

## Conclusions

This case illustrates the significant impact of systemic conditions such as hyperthyroidism on cardiac hemodynamics, particularly in patients with underlying structural heart disease like aortic stenosis. It also underscores the importance of evaluating and managing reversible systemic conditions, such as thyroid dysfunction, that can influence cardiac hemodynamics.
